# Association of Self-reported Primary Care Physician Tolerance for Uncertainty With Variations in Resource Use and Patient Experience

**DOI:** 10.1001/jamanetworkopen.2022.29521

**Published:** 2022-09-01

**Authors:** Arabella S. Begin, Michael K. Hidrue, Sara Lehrhoff, Inga T. Lennes, Katrina Armstrong, Jeffrey B. Weilburg, Marcela G. del Carmen, Jason H. Wasfy

**Affiliations:** 1 Department of Medicine, Massachusetts General Hospital, Boston; 2 Harvard Medical School, Boston, Massachusetts; 3 Massachusetts General Physicians Organization, Boston; 4 Columbia University Irving Medical Center, New York, New York; 5 Department of Psychiatry, Massachusetts General Hospital, Boston; 6 Division of Gynecologic Oncology, Department of Obstetrics and Gynecology and Reproductive Biology, Massachusetts General Hospital, Boston; 7 Cardiology Division, Department of Medicine, Massachusetts General Hospital, Boston

## Abstract

**Importance:**

Inappropriate variations in clinical practice are a known cause of poor quality and safety, with variations often associated with nonclinical factors, such as individual differences in cognitive processing. The differential response of physicians to uncertainty may explain some of the variations in resource use and patient experience.

**Objective:**

To examine the association of physician tolerance for uncertainty with variations in resource use and patient experience.

**Design, Setting, and Participants:**

This survey study linked physician survey data (May to June 2019), patient experience survey data (January 2016 to December 2019), and billing data (January 2019 to December 2019) among primary care physicians (PCPs) at Massachusetts General Hospital with at least 10 visits in 2019. The statistical analysis was performed in 2021.

**Main Outcomes and Measures:**

The analysis examined associations of PCP tolerance for uncertainty with the tendency to order diagnostic tests, the frequency of outpatient visits, hospital admissions, emergency department visits, and patient experience data (focused on physician communication and overall rating). A 2-stage hierarchical framework was used to account for clustering of patients under PCPs. Binary outcomes were modeled using a hierarchical logistic model, and count outcomes were modeled using hierarchical Poisson or negative binomial models. The analysis was adjusted for patient demographic variables (age, sex, and race and ethnicity), socioeconomic factors (payer and neighborhood income), and clinical comorbidities.

**Results:**

Of 217 included physicians, 137 (63.1%) were women, and 174 (80.2%) were adult PCPs. A total of 62 physicians (28.6%) reported low tolerance, 59 (27.2%) reported medium tolerance, and 96 (44.2%) reported high tolerance for uncertainty. Physicians with a low tolerance for uncertainty were less likely to order complete blood cell counts (odds ratio [OR], 0.66; 95% CI, 0.50-0.88), thyroid tests (OR, 0.67; 95% CI, 0.52-0.88), a basic metabolic profile (OR, 0.78; 95% CI, 0.60-1.00), and liver function tests (OR, 0.72; 95% CI, 0.53-0.99) than physicians with a high tolerance for uncertainty. Physicians who reported higher tolerance for uncertainty were more likely to receive higher patient experience scores for listening to patients carefully (OR, 0.65; 95% CI, 0.50-0.83) and higher overall ratings (OR, 0.80; 95% CI, 0.66-0.98) than physicians with medium tolerance. Conversely, no association was found between physician tolerance for uncertainty and patient outpatient visits, hospital admissions, or emergency department visits.

**Conclusions and Relevance:**

In clinical practice, identifying and effectively managing inappropriate variations and improving patient experience have proven to be difficult, despite increased attention to these issues. This study supports the hypothesis that physicians’ tolerance for uncertainty is associated with differences in resource use and patient experience. Whether enhancing physicians’ tolerance for uncertainty could help reduce unwarranted practice variations, improve quality and patient safety, and improve patient’s experience remains to be established.

## Key Points

**Question:**

Are variations in resource use and patient experience associated with physician self-reported tolerance for uncertainty?

**Findings:**

This survey study of 217 physicians found that primary care physicians with a low tolerance for uncertainty were less likely than those with a high tolerance for uncertainty to order diagnostic tests. Primary care physicians with less tolerance for uncertainty had worse patient experience scores than those with a high tolerance for uncertainty, although associations were not monotonic.

**Meaning:**

This study suggests that primary care physicians’ self-reported tolerance for uncertainty was associated with diagnostic test ordering and patient experience; however, because the causal links are not well defined, how tolerance for uncertainty could be potentially modified and what, if any, implications there are for health care quality and patient experience remain to be elucidated.

## Teaser

This survey study examines the association of physician tolerance for uncertainty with variations in resource use and patient experience.

## Introduction

Inappropriate variations in clinical practice are a known cause of poor quality care and safety, associated with poorer health outcomes, increased costs, disparities, and increased burden on the health system.^1,2^ For these reasons, a reduction in unwarranted clinical practice variations that cannot be explained by patient illness or patient preference is a central theme of quality improvement. Such variations in medical decision-making are often associated with clinician differences in both conscious and unconscious cognitive processing. One factor associated with cognitive processing is response to uncertainty.^3,4,5^ Tolerance for uncertainty represents the capacity to “close the gap between the normative ideal and the descriptive reality” of decision-making under uncertainty,^6^ and it can be defined as “the set of negative and positive psychological responses—cognitive, emotional, and behavioural—provoked by the conscious awareness of ignorance about particular aspects of the world.”^7^^(p62)^ A clinician’s tolerance for uncertainty is the balance between the positive and negative responses, which has been shown to be associated with the diagnostic process, with potential for diagnostic error that may effect patient outcomes.^8^ Findings on the association between tolerance for uncertainty and the use of health services have been mixed. On the one hand, low tolerance for uncertainty has been associated with increased tendencies to order tests,^9,10^ failure to comply with evidence-based guidelines,^11^ additional empirical treatment regimens,^12,13^ withholding of negative genetic test results,^14^ and fear of malpractice litigation and defensive practice.^15^ Conversely, some studies have shown that a lower tolerance for uncertainty may be associated with the lower use of tests,^16,17^ which may be associated with an unconscious suppression of uncertainty, leading to premature closure of the diagnostic reasoning process—the single most common phenomenon in misdiagnosis.^18^

Clinician tolerance for uncertainty also has conceptual causal links to patient experience. Studies have reported the undesirable effects of physicians’ communication of uncertainty, including heightened perceptions and feelings of vulnerability and avoidance of decision-making^19,20,21,22,23,24^ and negative patient perceptions (lack of confidence, low visit satisfaction, worry, or concern).^25,26,27,28,29,30,31^ Physicians who are intolerant of uncertainty are reluctant to disclose uncertainties to patients when making decisions, which can impede open, honest, and respectful communication.^32^ Inadequate management of uncertainty may cause unnecessary concern and distress to patients, risking undercutting the patient-physician relationship and decreasing trust.^33^ The inability to communicate uncertainty creates a false sense of certainty among patients, which can lead to substantial distrust when that certainty proves to be overstated.

To date, studies examining physicians’ tolerance for uncertainty have been mainly small scale, with many inconsistent findings, to our knowledge. To address this gap, we sought to examine the association of physician tolerance for uncertainty with variations in resource use and patient experience among primary care physicians (PCPs) at a large multispecialty academic physician practice organization.

## Methods

### Study Design, Population, and Setting

We performed a survey study linking near-comprehensive physician survey data, patient experience survey data, and hospital billing data among PCPs at Massachusetts General Hospital, the largest academic medical center in New England, to examine associations among physician tolerance for uncertainty, resource use, and patient experience. Primary care physicians were defined as adult or pediatric PCPs, regardless of type of board certification. This data set was well positioned to answer this analytic question owing to the near-complete capture of the variation data, along with a high response rate (approximately 90%) to our physician survey in the setting of an incentive payment. The Mass General Brigham institutional review board approved this study. The Partners Human Research Committee determined that the project met the criteria for institutional review board exemption because the research was limited to the use of survey data, was not subject to US Food and Drug Administration regulations, and there was no more than minimal risk to study participants. Completion of the survey was considered implied consent of participation. All data used were strictly anonymized; only a research coordinator had access to the file linking responses to identifiers. Physicians were asked to consent to survey participation on the cover letter inviting them to participate. All methods for the survey are in compliance with the American Association for Public Opinion Research (AAPOR) reporting guideline for survey studies.^34^

### Survey Instrument and Variables

Information on physician tolerance for uncertainty was collected from the 2019 biennial Massachusetts General Physicians Organization (MGPO) survey. The main goals of the MGPO survey were to assess physician well-being, direct priorities regarding funding of practices, evaluate department and division chiefs, and better understand the functioning of the clinical enterprise. Tolerance for uncertainty was measured using the single item, “I find the uncertainty involved in patient care disconcerting,” adapted from the 15-item Physicians’ Reaction to Uncertainty Scale, developed by Gerrity et al.^35^ This single item has been shown to stratify tolerance for uncertainty among physicians,^36,37,38^ and it is often used in surveys such as ours addressing multiple content areas within space constraints where use of the full scale is limited by length. The score for this single item ranges from 1 to 5, with 5 signifying greatest discomfort from uncertainty (or lowest tolerance for uncertainty) and 1 signifying least discomfort from uncertainty (or greatest tolerance for uncertainty). The extent to which participants agreed with the statement was reduced to 3 categories: low tolerance (strongly agree or moderately agree), medium tolerance (neither agree nor disagree), and high tolerance (moderately disagree or strongly disagree). The responses to this question along with some physician characteristics (gender and race and ethnicity) were extracted for merging with billing data on resource use and patient survey responses. Data on physician race were self-reported from categories including American Indian or Alaska Native, Native Hawaiian, Asian, Black, White, other, and prefer not to say. Data on physician ethnicity were self-reported from categories including Hispanic, non-Hispanic, and prefer not to say. We speculate that the “other” category may include physicians from parents of different races or physicians who self-identify as a race other than the provided categories, such as Middle Eastern and North African. Data on race and ethnicity were collected to assess equity among physicians at our organization.

The MGPO collects data on patient experience from a random sample of patients after outpatient visits through the Clinician and Group Consumer Assessment of Healthcare Providers and Systems Survey.^39^ For this analysis, we focused on the physician communication and overall physiclinician rating domains of this survey: “physician explained things in a way that is easy to understand” (MD Explain); “physician listened carefully to patient” (MD Listen); “physician showed respect for what the patient had to say” (MD Respect); “physician spent enough time with patient” (MD Time); and “overall, how do you rate this physician” (MD Rate). To increase the number of responses per physician, we used data collected from 2016 through 2019. When a patient completed multiple surveys during this period, we used the latest survey. The rest of the data are extracted from practice billing records, including diagnostic test orders, outpatient visits, emergency department (ED) visits, patients’ demographic characteristics, and patients’ comorbidity indicators. A detailed description of outcome variables is provided in the eMethods in the Supplement.

### Sensitivity Analysis

Some of the data on patient experience came from years prior to the year when physician tolerance for uncertainty was measured (2019). Because tolerance for uncertainty improves with experience, one can argue that the physician level of tolerance in those years might have been different, potentially creating noise in our results. To test for such a possibility, we performed a sensitivity analysis by using only 2019 responses and specifying a single-level model (rather than hierarchical model). This sensitivity analysis did not change our main findings from the full model.

### Statistical Analysis

Standard descriptive statistics were used to summarize data and compare the distributions among the 3 categories of physician tolerance for uncertainty. Because the degree of tolerance for uncertainty is ordinal in nature, we used trend-based tests to assess the association of physician tolerance for uncertainty with other variables. For categorical variables, we used the Cochran-Armitage test and the Cochran-Mantel-Haenszel test for row mean scores, as appropriate. For continuous variables, we used the Jonckheere-Terpstra test. For modeling and risk adjustment, we used a 2-stage hierarchical model with physicians as a random effect to account for clustering of patients within physicians. Binary outcomes were modeled using random-effect logistic regression, and count data outcomes were modeled using random-effect Poisson regression. For models of patient experience, we adjusted for patient age, patient-PCP gender (same vs different), patient-PCP race and ethnicity (same vs different), educational level, self-reported health status, length of relationship with physician, and visit year. For the rest of the models, we adjusted for demographic characteristics (age, gender, and race and ethnicity), socioeconomic variables (payer type and zip code income), comorbidity indicators, and physician class (pediatric vs adult). All *P* values were from 2-sided tests, and results were deemed statistically significant at *P* < .05. Regression results are reported as odds ratios (ORs) or rate ratios depending on the nature of the outcome measure. Analyses were performed using SAS, version 9.4 (SAS Institute Inc).

## Results

### Physician Tolerance for Uncertainty and Physician Characteristics

There were 243 PCPs with at least 10 visits in 2019, and 217 (89.3%) completed the physician survey; of those, 137 (63.1%) were women, and 174 (80.2%) were adult PCPs (Table 1). A total of 62 PCPs (28.6%) reported a low tolerance for uncertainty, 59 (27.2%) reported a medium tolerance for uncertainty, and 96 (44.2%) reported a high tolerance for uncertainty. There was no association between physicians’ tolerance for uncertainty and gender (low tolerance: 41 of 137 female PCPs [29.9%] vs 20 of 74 male PCPs [27.0%]; medium tolerance: 32 of 137 female PCPs [23.4%] vs 24 of 74 male PCPs [32.4%]; high tolerance: 64 of 137 female PCPs [46.7%] vs 30 of 74 male PCPs [40.5%]; *P* = .18) or PCP type (low tolerance: 50 of 174 [28.7%] with adult patients vs 12 of 43 [27.9%] with pediatric patients; medium tolerance: 48 of 174 [27.6%] with adult patients vs 11 of 43 [25.6%] with pediatric patients; high tolerance: 76 of 174 [43.7%] with adult patients vs 20 of 43 [46.5%] with pediatric patients; *P* = .80). However, physicians with a lower tolerance for uncertainty had significantly fewer years since training than physicians with a high tolerance for uncertainty (median, 16 years [IQR, 6-25 years] vs 20 years [IQR, 10-30 years]; *P* = .04) as well as a slightly larger panel size (median, 796 patients [IQR, 576-1062 patients] vs 718 patients [IQR, 490-1047 patients]; *P* = .17).

**Table 1.** Association of Physician Tolerance for Uncertainty With PCP and Patient Characteristics

Characteristic	Physician degree of tolerance for uncertainty, No./total No. (%)			*P* value
	Low (n = 62)	Medium (n = 59)	High (n = 96)	
**PCP characteristics**				
PCP experience, median (IQR), y	16 (6-25)	19 (7-25)	20 (10-30)	.04
PCP panel size, median (IQR), No. patients	796 (576-1062)	786 (576-1091)	718 (490-1047)	.17
PCP gender^a^				
Female	41/137 (29.9)	32/137 (23.4)	64/137 (46.7)	.18
Male	20/74 (27.0)	24/74 (32.4)	30/74 (40.5)	
Preferred not to specify	1/6 (16.7)	3/6 (50.0)	2/6 (33.3)	
PCP type^a^				
Adult	50/174 (28.7)	48/174 (27.6)	76/174 (43.7)	.80
Pediatric	12/43 (27.9)	11/43 (25.6)	20/43 (46.5)	
**Patient characteristics**				
Age group, y				
<50	20 459/35 393 (57.8)	16 881/33 518 (50.4)	22 557/48 733 (46.3)	<.001
50-69	9812/35 393 (27.7)	10 739/33 518 (32.0)	16 719/48 733 (34.3)	
≥70	5122/35 393 (14.5)	5898/33 518 (17.6)	9457/48 733 (19.4)	
Gender				
Male	15 845/35 393 (44.8)	15 820/33 518 (47.2)	20 114/48 733 (41.3)	<.001
Female	19 548/35 393 (55.2)	17 698/33 518 (52.8)	28 619/48 733 (58.7)	
Ethnicity				
Hispanic	6780/35 393 (19.2)	3321/33 518 (9.9)	5949/48 733 (12.2)	<.001
Non-Hispanic	28 613/35 393 (80.8)	30 197/33 518 (90.1)	42 784/48 733 (87.8)	
Race				
Asian	2710/35 393 (7.7)	2962/33 518 (8.8)	3179/48 733 (6.5)	<.001
Black	2705/35 393 (7.6)	2041/33 518 (6.1)	3104/48 733 (6.4)	
White	21 915/35 393 (61.9)	23 911/33 518 (71.3)	34 689/48 733 (71.2)	
Other^b^	8063/35 393 (22.8)	4604/33 518 (13.7)	7761/48 733 (15.9)	
Payer group				
BCBS	10 547/35 393 (29.8)	11 280/33 518 (33.7)	15 305/48 733 (31.4)	<.001
Other commercial	10 987/35 393 (31.0)	10 691/33 518 (31.9)	14 684/48 733 (30.1)	
Medicaid	5721/35 393 (16.2)	3671/33 518 (11.0)	6111/48 733 (12.5)	
Medicare	4476/35 393 (12.7)	5021/33 518 (15.0)	8110/48 733 (16.6)	
Missing	3662/35 393 (10.4)	2855/33 518 (8.5)	4523/48 733 (9.3)	
Income, $				
<65 000	10 413/35 393 (29.4)	7540/33 518 (22.5)	10 238/48 733 (21.0)	<.001
65 000-96 000	8691/35 393 (24.6)	7826/33 518 (23.4)	12 089/48 733 (24.8)	
97 000-120 000	9147/35 393 (25.8)	9457/33 518 (28.2)	13 495/48 733 (27.7)	
>120 000	7142/35 393 (20.2)	8695/33 518 (25.9)	12 911/48 733 (26.5)	

Abbreviations: BCBS, Blue Cross Blue Shield; PCP, primary care physician.

^a^ The row totals are the denominators for these PCP characteristics.

^b^ Includes American Indian or Alaska Native, Native Hawaiian or Other Pacific Islander, and those who declined to provide race information.

### Physician Tolerance for Uncertainty and Patient Characteristics

Physicians with a low tolerance for uncertainty tended to have a higher proportion of patients younger than 50 years than did physicians with a high tolerance for uncertainty (20 459 of 35 393 [57.8%] vs 22 557 of 48 733 [46.3%]; *P* < .001) as well as patients with incomes lower than $65 000 (10 413 of 35 393 [29.4%] vs 10 238 of 48 733 [21.0%]; *P* < .001) (Table 1). The association of physician tolerance for uncertainty with patient age was overall similar when we excluded pediatric patients from the analysis. For the adult population, the proportion of patients younger than 50 years was 43.6% (11 536 of 26 470) among physicians with low tolerance for uncertainty and 34.0% (13 461 of 39 637) among physicians with high tolerance for uncertainty (*P* < .001).

### Physician Tolerance for Uncertainty and Diagnostic Test Ordering

In unadjusted comparisons, physicians with a low tolerance for uncertainty were less likely than physicians with a high tolerance to order complete blood cell counts (CBCs) (8852 of 35 393 [25.0%] vs 16 929 of 48 733 [34.7%]; *P* < .001), CBCs with differential (3658 of 35 393 [10.3%] vs 5635 of 48 733 [11.6%]; *P* < .001), thyroid tests (6229 of 35 393 [17.6%] vs 12 676 of 48 733 [26.0%]; *P* < .001), basic metabolic profiles (BMPs) (11 870 of 35 393 [33.5%] vs 20 413 of 48 733 [41.9%]; *P* < .001), lipid tests (11 732 of 35 393 [33.2%] vs 18 939 of 48 733 [38.9%]; *P* < .001), liver function tests (LFTs) (9166 of 35 393 [25.9%] vs 16 378 of 48 733 [33.6%]; *P* < .001), and high-cost imaging (2573 of 35 393 [7.3%] vs 4014 of 48 733 [8.2%]; *P* < .001) (Table 2).

**Table 2.** Unadjusted Association of Physician Tolerance for Uncertainty With Selected Outcomes

Outcome	Sample size	Physician degree of tolerance for uncertainty, No. (%)			*P* value^a^
		Low	Medium	High	
Patient experience^b^					
MD					
Explain	8531	2155/2365 (91.1)	2166/2398 (90.3)	3492/3768 (92.7)	.02
Listen	8531	2166/2365 (91.6)	2159/2398 (90.0)	3524/3768 (93.5)	.001
Respect	8531	2237/2365 (94.6)	2252/2398 (93.9)	3571/3768 (94.8)	.63
Time	8531	2088/2365 (88.3)	2105/2398 (87.8)	3372/3768 (89.5)	.10
Rate	8531	1932/2365 (81.7)	1954/2398 (81.5)	3231/3768 (85.8)	<.001
Patients with diagnostic tests^c^					
CBC	117 644	8852/35 393 (25.0)	11 192/33 518 (33.4)	16 929/48 733 (34.7)	<.001
CBC with differential	117 644	3658/35 393 (10.3)	3823/33 518 (11.4)	5635/48 733 (11.6)	<.001
Thyroid	117 644	6229/35 393 (17.6)	7801/33 518 (23.3)	12 676/48 733 (26.0)	<.001
BMP	117 644	11 870/35 393 (33.5)	14 058/33 518 (41.9)	20 413/48 733 (41.9)	<.001
Lipid	117 644	11 732/35 393 (33.2)	13 230/33 518 (39.5)	18 939/48 733 (38.9)	<.001
LFT	117 644	9166/35 393 (25.9)	10 056/33 518 (33.6)	16 378/48 733 (33.6)	<.001
High-cost imaging	117 644	2573/35 393 (7.3)	2723/33 518 (8.1)	4010/48 733 (8.2)	<.001
Outpatient visits					
PCP visits, median (IQR)	117 644	1 (1-2)	1 (1-2)	1 (1-2)	.41
Specialist visits, median (IQR)	117 644	1 (0-3)	1 (0-3)	1 (0-3)	<.001
Acute care^c^					
ED use	117 644	7558/35 393 (21.4)	6048/33 518 (18.0)	9434/48 733 (19.4)	<.001

Abbreviations: BMP, basic metabolic profile; CBC, complete blood cell count; ED, emergency department; LFT, liver function test; MD, medical doctor; PCP, primary care physician.

^a^ Trend-based tests are used to test association of outcomes with physician level of tolerance for uncertainty. For categorical outcomes with 2 levels, we used the Cochran-Armitage test, and for continuous outcomes, we used the Jonckheere-Terpstra test.

^b^ For patient experience outcomes, except for MD Time, physicians with a medium tolerance for uncertainty have lower scores than physicians with a low tolerance for uncertainty. However, pairwise comparisons show that the difference in top score between physicians with a medium tolerance and a low tolerance for uncertainty is not statistically significant.

^c^ Diagnostic tests and ED admissions are all specified as binary outcomes. We initially considered modeling them as count outcomes, but most patients had either 0 or 1 value. The proportions of patients with more than 1 test per year were 9.4% (11 093 of 117 644) for BMP, 6.0% (7100 of 117 644) for LFTs, 5.1% (5965 of 117 644) for CBC, 3.9% (4533 of 117 644) for lipid tests, 1.8% (2139 of 117 644) for high-cost imaging, and 1.6% (1895 of 117 644) for CBC with differential. Similarly, the proportion of patients with more than 1 visit was 6.4% (7493 of 117 644) for ED visits.

Adjusting for case-mix factors, we found that physicians with a low tolerance for uncertainty were less likely than physicians with a high tolerance to order CBCs (OR, 0.66; 95% CI, 0.50-0.88), thyroid tests (OR, 0.67; 95% CI, 0.52-0.88), BMPs (OR, 0.78; 95% CI, 0.60-1.00), and LFTs (OR, 0.72; 95% CI, 0.53-0.99) (Table 3). Differences in test ordering tendency between physicians with a low tolerance for uncertaintly and physicians with a high tolerance for uncertainty were not statistically significant for ordering CBCs with differential, lipid tests, and high-cost imaging. Lastly, differences in test ordering tendencies between physicians with a medium tolerance for uncertainty and physicians with a high tolerance for uncertainty were not statistically significant.

**Table 3.** Risk-Adjusted Association of Physician Tolerance for Uncertainty With Selected Outcomes^a^

Outcome	Odds ratio or rate ratio (95% CI)^b^	
	Medium vs high tolerance (reference)	Low vs high tolerance (reference)
Patient experience, odds ratio (95% CI)		
MD		
Explain	0.79 (0.60-1.04)	0.88 (0.67-1.16)
Listen	0.65 (0.50-0.83)	0.79 (0.61-1.02)
Respect	0.91 (0.68-1.22)	1.05 (0.79-1.39)
Time	0.87 (0.66-1.15)	0.92 (0.73-1.16)
Rate	0.80 (0.66-0.98)	0.85 (0.68-1.06)
Diagnostic tests, odds ratio (95% CI)		
CBC	0.91 (0.68-1.23)	0.66 (0.50-0.88)
CBC with differential	0.96 (0.66-1.40)	1.07 (0.76-1.52)
Thyroid	0.87 (0.68-1.12)	0.67 (0.52-0.88)
BMP	1.02 (0.80-1.29)	0.78 (0.60-1.00)
Lipid	1.05 (0.85-1.30)	0.91 (0.73-1.14)
LFT	0.79 (0.57-1.09)	0.72 (0.53-0.99)
High-cost imaging	0.99 (0.85-1.19)	1.01 (0.86-1.19)
Outpatient visits, rate ratio (95% CI)		
PCP	1.02 (0.96-1.08)	1.00 (0.94-1.06)
Specialist	1.01 (0.95-1.06)	1.02 (0.96-1.07)
Acute care, odds ratio (95% CI)		
ED	0.94 (0.84-1.05)	1.08 (0.95-1.22)

Abbreviations: BMP, basic metabolic profile; CBC, complete blood cell count; ED, emergency department; LFT, liver function test; MD, medical doctor; PCP, primary care physician.

^a^ In addition to PCP level of tolerance, models also adjusted for the following variables (to save space, we have reported only estimates of tolerance for uncertainty): (1) patient experience: patient-PCP gender (same vs different), patient-PCP race (same vs different), visit year, patient age, length of relationship with PCP, self-reported health status, and educational level; (2) diagnostic tests: PCP class (adult vs pediatric), race, payer group, zip code median income, number of PCP visits, number of specialist visits, and presence of the following comorbidities: chronic obstructive pulmonary disease, chronic kidney disease, congenital heart failure, uncomplicated diabetes, complicated diabetes, mild liver disease, and peripheral vascular disease; (3) outpatient visits: PCP class (adult vs pediatrics), gender, age, race, payer group, zip code median income, and the presence of the following comorbidities: chronic obstructive pulmonary disease, chronic kidney disease, congenital heart failure, uncomplicated diabetes, complicated diabetes, mild liver disease, and peripheral vascular disease; (4) ED admissions: PCP class (adult vs pediatrics), gender, age, race, payer group, zip code median income, and the presence of the following comorbidities: chronic obstructive pulmonary disease, chronic kidney disease, congenital heart failure, uncomplicated diabetes, complicated diabtes, mild liver disease, and peripheral vascular disease.

^b^ These results are based on a 2-stage hierarchical model with random effect and random intercept for PCPs. Outcomes for patient experience, diagnostic tests, and admissions are based on a hierarchical logistic model, and their results are reported as odds ratios. Outcomes for outpatient visits are based on hierarchical Poisson models, and regression results are reported as rate ratio.

### Physician Tolerance for Uncertainty and Outpatient Visits

The median number of PCP visits per year were the same across all 3 groups of physicians (median, 1 [IQR, 1-2]). Similarly, the median number of visits to specialists were similar among the patients of the 3 physician groups (median, 1 [IQR, 0-3]) (Table 2). After adjusting for risk factors, we found no association between physician’s level of tolerance for uncertainty and their patients’ visits to PCP or specialty offices (Table 3).

### Physician Tolerance for Uncertainty and ED Visits

In unadjusted comparisons, we found that patients whose PCP had a lower tolerance for uncertainty were more likely to visit the ED than patients whose PCP had a medium or high tolerance for uncertainty (7558 of 35 393 [21.4%] vs 6048 of 33 518 [18.0%] and 9434 of 48 733 [19.4%], respectively; *P* < .001) (Table 2). After adjustment for risk factors, these differences were not statistically significant (Table 3). Finally, the likelihood of 30-day readmission was not associated with PCPs’ tolerance for uncertainty in both adjusted and unadjusted models (Table 2 and Table 3).

### Physician Tolerance for Uncertainty and Patient Experience

Table 2 presents unadjusted associations between physician tolerance for uncertainty and our outcomes of interest. Physicians with a high tolerance for uncertainty were more likely than those with medium and low tolerance for uncertainty to receive a top score on MD Explain (3492 of 3768 [92.7%] vs 2166 of 2398 [90.3%] and 2155 of 2365 [91.1%], respectively; *P* = .02), MD Listen (3524 of 3768 [93.5%] vs 2159 of 2398 [90.0%] and 2166 of 2365 [91.6%], respectively; *P* = .001), and MD Rate (3231 of 3768 [85.8%] vs 1954 of 2398 [81.5%] and 1932 of 2365 [81.7%], respectively; *P* < .001). There was no significant difference in the scores for MD Respect (high, 3571 of 3768 [94.8%] vs medium, 2252 of 2398 [93.9%] vs low, 2237 of 2365 [94.6%]; *P* = .63) and MD Time (high, 3372 of 3768 [89.5%] vs medium, 2105 of 2398 [87.8%] vs low, 2088 of 2365 [88.3%]; *P* = .10).

Table 3 presents risk-adjusted parameter estimates for all our measured outcomes. Physicians with a higher tolerance for uncertainty were more likely than physicians with a medium tolerance for uncertainty to listen to patients carefully (MD Listen; OR, 0.65; 95% CI, 0.50-0.83) and to receive higher overall ratings (MD Rate; OR, 0.80; 95% CI, 0.66-0.98). These associations, however, were not demonstrated for physicians with a low tolerance for uncertainty compared with physicians with a high tolerance for uncertainty (MD Listen: OR, 0.79; 95% CI, 0.61-1.02; MD Rate: OR, 0.85; 95% CI, 0.68-1.06).

## Discussion

This study adds new knowledge by examining associations between PCP tolerance for uncertainty and variations in resource use and patient experience. We found that PCPs who reported a lower tolerance for uncertainty ordered fewer diagnostic tests (specifically CBCs, thyroid tests, BMPs, and LFTs). Physicians who reported a lower tolerance for uncertainty also had worse patient-reported survey scores for listening carefully and for overall rating of patient experience, although this association was not monotonic and was less clearly established. We also found that physicians with a lower tolerance for uncertainty had fewer years since training and higher proportions of patients with a more vulnerable socioeconomic status than physicians with a high tolerance for uncertainty. Given that this research question requires detailed and complete information about physicians’ clinical practice and associated patient experience, it is likely unanswerable using information from large national data sets. Although this is a single-center study, the opportunity to compare the degrees of physician tolerance for uncertainty with data on variations is rare, especially with a data set that has near-complete capture.

In contrast to prior research finding that a low tolerance for uncertainty was associated with increased test-ordering tendencies,^9,10^ we found that physicians with a low tolerance for uncertainty were less likely to order diagnostic tests, risking premature closure on the diagnostic reasoning process and an increased chance of diagnostic error and downstream negative ramifications for the patient and the health care system.^18^ These prior results were derived in the ED setting, so we speculate that our results may differ because we studied PCPs. Both underuse and overuse of diagnostic testing can be associated with low-quality care. As such, the implications of our findings about the association between tolerance for uncertainty and overall health care quality are uncertain.

This study builds on earlier research evaluating the tolerance for uncertainty among health care professionals. Tolerance for uncertainty has been associated with various practice-related attitudes of medical students, with studies showing that students with a lower tolerance for uncertainty showed a more negative orientation toward patients’ psychological problems, more Machiavellianism, a preference for a structured work environment,^40,41^ more negative attitudes toward the underserved,^42^ and bias against those who have alcohol use disorder.^43^ We found differences in some patient experience measures (listening and overall rating) but not others (explaining, respect, and time spent), and the demonstrated associations with listening and overall rating were not monotonic. Physicians with a medium tolerance for uncertainty had worse listening and overall rating scores than physicians with a high tolerance for uncertainty. These associations may be complex, however, because physicians with a low tolerance for uncertainty demonstrated a statistically insignificant trend toward lower ratings on only those 2 measures. Disclosing and discussing uncertainty have been recognized to be the 2 most challenging elements of risk communication.^44^ Although physicians often worry that admitting uncertainty will lead to loss of patient confidence, it has been suggested that appropriate expressions of uncertainty can lead to stronger physician-patient relationships.^45^ One study found that when PCPs used direct expressions of uncertainty, such as “I don’t know” or “It’s not clear,” there were higher levels of positive talk, patient engagement, and patient satisfaction.^46^ It may be that physicians with a higher tolerance for uncertainty were able to communicate uncertainty better to patients, which was associated with higher overall ratings and a feeling of being listened to more carefully. By discussing uncertainty with patients, whether about diagnosis, prognosis, or treatment and management options, physicians may find a reduction in their stress and anxiety through sharing decision-making responsibility. A patient’s values and preferences can often guide treatment choices when otherwise the best means of proceeding would be uncertain.

Our results suggest that responses to uncertainty may be associated with variations in resource use and patient experience. There is much evidence to suggest that tolerance for uncertainty is a state, not a trait, and therefore amenable to change through an educational and experiential process,^47^ although there is likely an association with inherited personality traits and environmental influences that predispose individuals to specific psychological responses. Particular attention likely needs to be paid to those with less experience, with senior colleagues acting as role models to normalize the experience of uncertainty. Understanding and acknowledging uncertainty and acquiring proper coping strategies are now regarded as core clinical competencies for medical graduates and trainees in the UK, US, Australia, and much of Europe,^48,49,50,51,52,53,54^ but there is still much about tolerance for uncertainty that is not understood. Further studies are needed that concentrate on the associations between reactions to uncertainty and patient outcomes to establish to what extent uncertainty is associated with quality of care. More work is needed to define the circumstances and communication strategies for uncertainty, exploring the fundamental questions about how people process, interpret, and respond to various types of uncertainty inherent in clinical decisions and the diagnostic reasoning process.

### Limitations

This study has several limitations. First, our results were subject to the inherent reporting biases that often occur in survey studies. However, because this was a retrospective analysis, any social desirability biases were minimized because survey participants were unaware of the specific hypothesis of this study and all data were collected in a deidentified and confidential manner. Owing to space constraints, we used a single-item self-reported measure to assess tolerance for uncertainty. This item is unvalidated, although it has been shown to stratify the degrees of tolerance for uncertainty in physicians.^36,37,38^ This single question has been shown to have a good spread and to be a useful measure when space constraints prevent the whole 15-item survey from being used.^36,37,38^ However, conceptual definitions of uncertainty and tolerance vary, and different physicians may have interpreted the question differently. The variation data were collected separately without risk of reporting bias. Our response rate of 89.3% is a robust response, increasing the accuracy of the data and minimizing selection bias. Second, our results may not be generalizable beyond PCPs, although there is no reason to postulate that these findings would be unique to primary care. Nevertheless, further studies are needed to confirm our findings in other hospital and academic settings. Third, as a single-center study, it is unclear the extent to which we can generalize these findings to other settings. However, we do think that, given the unusual lack of missing data in our survey, the internal validity of our results are better than a multisite survey with missing data. Fourth, we demonstrated improvements in some patient experience measures for physicians with a high tolerance for uncertainty relative to those with a medium tolerance for uncertainty, but these differences were not apparent for physicians with a low level of tolerance for uncertainty. These associations may be complex and nonmonotonic, or a larger data set with more statistical power might demonstrate a monotonic association. Fifth, although we demonstrated an association between lower self-reported tolerance for uncertainty and fewer tests ordered, we cannot know from these data how frequently these tests were ordered in guideline-adherent or appropriate ways. Sixth, owing to the observational design of our study, we were careful to test only for associations and do not draw conclusions about causality from our findings alone. We do not know if interventions to improve tolerance for uncertainty among physicians would be effective, nor if they would improve clinical quality and outcomes. However, our results at least raise the hypothesis that efforts to improve tolerance for uncertainty in medical training and practice may improve patient satisfaction and could increase ordering of tests.

## Conclusions

Identifying and effectively managing inappropriate variations in clinical practice have proven to be difficult. This study supports the hypothesis that physicians’ tolerance for uncertainty is associated with differences in resource use and patient experience and may, therefore, be associated with some of the variations, lending support to improving the management of uncertainty. Enhancing physician tolerance for uncertainty may help reduce unwarranted clinical practice variations and may also improve the patient experience by enhancing communication and satisfaction. By shifting the culture of medicine to acknowledge and openly discuss uncertainty—with colleagues and patients—empathetic, positive, and partnering relationships can be established that may bolster trust and increase patient engagement and comfort, improving communication, patient safety, and physician well-being.

## Notes

Supplement. **eMethods.** Detailed Description of Outcome Variables
