# Supplementary material for: Association of Self-reported Primary Care Physician Tolerance for Uncertainty With Variations in Resource Use and Patient Experience
Source: JAMA Netw Open. 2022 Sep 1;5(9):e2229521. doi: 10.1001/jamanetworkopen.2022.29521 (PMC9437748; doi:10.1001/jamanetworkopen.2022.29521)
Supplement: Supplement. — eMethods. Detailed Description of Outcome Variables [file jamanetwopen-e2229521-s001.pdf]

Supplementary Online Content

Begin AS, Hidrue MK, Lehrhoff S, et al. Association of self-reported primary care physician tolerance for uncertainty with variations in resource use and patient experience. *JAMA Netw Open.* 2022;5(9):e2229521. doi:10.1001/jamanetworkopen.2022.29521

eMethods. Detailed Description of Outcome Variables

This supplementary material has been provided by the authors to give readers additional information about their work.

© 2022 Begin AS et al. *JAMA Network Open.*

eMethods. Detailed Description of Outcome Variables

Diagnostic Tests: We chose six labs (complete blood count (CBC), CBC with differentials, thyroid, basic metabolic panel (BMP), LIPID, and liver function test (LFT)) and one imaging metric (high-cost imaging, which includes ultrasound, magnetic resonance, computed tomography, positron emission tomography and positron emission tomography-computed tomography) for this category. We specified each test as a binary outcome and modeled the tendency to order a test.

Outpatient Visits: We examined number of PCP visits and number of specialist visits per year. PCP visits include visits to the patient’s PCP only whereas specialist visits, which are included as a proxy for PCP referrals, include visits to any specialist. Both number of PCP visits and number of specialist visits are modeled as count data variables.

Emergency Room Admissions: ED visit includes only ED visits discharged home. ED visits admitted to the hospital (either through observation or inpatient status) are not included as ED visits. For simplicity, we specified ED visit as binary outcome.

Patient Experience: We considered the following five items from the CG-CAHPS survey: “provider explained things in a way that is easy to understand” (MD Explain); “provider listened carefully to patient” (MD Listen); “provider showed respect for what the patient had to say” (MD Respect); “provider spent enough time with patient” (MD Time); “overall, how do you rate this provider” (MD Rate). The first four items represent provider communications. Each item has four responses: never, sometimes, usually, and always. These items are mostly analyzed as a composite score. However, since we are interested in understanding how physician tolerance of uncertainty affects different aspects of a patient

© 2022 Begin AS et al. *JAMA Network Open.*

experience, we analyzed each item separately. Each item is scored as top-box score (1 if the patient responded ‘always’; 0 otherwise). The overall provider rate has 10 responses and the top 2 scores (9 and 10) are considered as top-box score. For this analysis, we excluded pediatric PCPs because the number of responses was low.

Risk Adjustment Covariates: We collected a wide range of covariates from the hospital’s billing data, patient experience survey, and physician wellbeing survey. For patient experience models, we adjusted for patient age, patient-PCP gender (same vs different), patient-PCP race (same vs different), level of education, self-reported health status, length of relationship with provider, and visit year. These variables came from the patient experience survey and physician wellbeing survey. For the rest of the models, we adjusted for demographics (age, gender, race, and ethnicity), socio-economic variables (payor type and zip code income), comorbidity indicators, and provider class (pediatric vs adult). Comorbidities used include chronic obstructive pulmonary disease, chronic kidney disease, congenital heart failure, diabetes, liver disease, and peripheral vascular disease. We included comorbidities with incidence rates of 2% or above.

© 2022 Begin AS et al. *JAMA Network Open.*
